# Additive Soft Matter Design by UV-Induced Polymer Hydrogel Inter-Crosslinking

**DOI:** 10.3390/gels8020117

**Published:** 2022-02-14

**Authors:** Talika A. Neuendorf, Niclas Weigel, Michelle Vigogne, Julian Thiele

**Affiliations:** Leibniz-Institut für Polymerforschung Dresden e. V., Hohe Straße 6, 01069 Dresden, Germany; neuendorf@ipfdd.de (T.A.N.); weigel@ipfdd.de (N.W.); vigogne@ipfdd.de (M.V.)

**Keywords:** hydrogel, assembly, additive manufacturing, photopolymerization, crosslinking, building blocks, droplet microfluidics

## Abstract

In recent years, stimuli-responsive hydrogels have gained tremendous interest in designing complex smart 4D materials for applications ranging from biomedicine to soft electronics that can change their properties on demand over time. However, at present, a hydrogel’s response is often induced by merely a single stimulus, restricting its broader applicability. The controlled hierarchical assembly of various hydrogel building blocks, each with a tailored set of mechanical and physicochemical properties as well as programmed stimulus response, may potentially enable the design and fabrication of multi-responsive polymer parts that process complex operations, like signal routing dependent on different stimuli. Since inter-connection stability of such building blocks directly accompanies the transmission of information across building blocks and is as important as the building property itself to create complex 4D materials, we provide a study on the utility of an inter-crosslinking mechanism based on UV-induced 2,3-dimethylmaleimide (DMMI) dimerization to inter-connect acrylamide-based and *N*-isopropylacrylamide-based millimeter-sized cubic building blocks, respectively. The resulting dual-crosslinked assemblies are freestanding and stable against contraction–expansion cycles in solution. In addition, the approach is also applicable for connecting microfluidically fabricated, micrometer-sized hydrogel spheres, with the resulting assemblies being processable and mechanical stable, likewise resisting contraction–expansion in different solvents, for instance.

## 1. Introduction

The concept of controlled hierarchical assembly of building blocks is a well-known theme in our lives, e.g., when constructing stone walls, playing with interlocking plastic bricks, or looking at cellular organization in organisms. The idea of utilizing small building blocks with different colors or shapes to build up larger objects enables the constructor to tailor individually local and global properties, shape, size, and appearance of the final part. The transfer of this concept to the field of materials science and additive manufacturing to create assemblies of building blocks exhibiting distinct properties and yielding complex multifunctional materials still remains a challenge. A key material basis for the design of these building blocks are hydrogels, which are widely used in rather different areas, like biomedicine [1] or sensor systems [2], where they exhibit a wide range of properties being magnetic [3], electrically conductive [4], or thermo-responsive [5]. Several groups have already developed various assembly techniques and inter-connection concepts utilizing hydrogel-based building blocks towards multifunctional materials. One of the first works dates back to 2008, investigating the directed assembly of cell-laden microscopic hydrogels [6]. The authors fabricated polyethylene glycol methacrylate-based hydrogels by a photomask approach to yield cubic structures with approximate dimensions of 400 µm × 400 µm × 150 µm. After immersion in mineral oil, the cubes were assembled by mechanical agitation utilizing a pipette tip, followed by inter-crosslinking of the cubic structures to yield different assembly geometries. The authors further demonstrated the utility of their approach by assembling cross-shaped and rod-shaped hydrogels in a lock-and-key design. A more recent example of guided assembly of hydrogel-based systems is the work of Downs et al., where the authors formed microgel assemblies in oil by deposition of droplets containing various pre-polymer solutions [7]. Subsequent UV exposure resulted in the rupture of the droplet-stabilizing surfactant interface and partial coalescence of the droplets while radical polymerization simultaneously formed stable and connected hydrogel structures. With that, they were able to implement and combine different stimuli-responsive properties inside the microgel structures, e.g., to utilize them as a magnetically and thermo-responsive gripper for cargo transport. Further examples of guided building block assemblies are based on multi-nozzle extrusion [8], sliding-based transport utilizing micro-robots [9], building block alignment via photo-patterning [10], and guided assembly inside specialized microfluidic devices [11,12]. The afore-discussed approaches rather focus on controlling building block deposition than on their inter-connectivity, with none of them considering the utilization of a separate connection mechanism, but instead mostly relying on unsaturated moieties of the respective base material to inter-connect the hydrogel structures.

While these inter-connection concepts are rather straightforward and easy to apply, we believe it is beneficial to introduce additional reactive moieties only contributing to the connection mechanism. With that, we aim for precisely controlling the mechanical stability of multi-particle assemblies, so-called supragel constructs and clearly separate building block formation and building block inter-crosslinking. In this regard, the combination of different base materials has previously been utilized for dual crosslinking of microgels. Exemplarily, poly(*N*-isopropylacrylamide)-based supramolecular hydrogels have been produced exploiting adamantane and β-cyclodextrin moieties to reversibly connect microgels based on a host–guest mechanism [13]. Another example is the utilization of reversible disulfide bonding based on *N*,*N*’-bis(acryloyl)cystamine to inter-connect thermo-responsive microgels consisting of poly(*N*-isopropylmethacrylamide) [14]. Other work has focused on irreversible microgel crosslinking by introducing additional vinyl groups through copolymerization of the acrylic base material with glycidyl methacrylate [15] or 2-hydroxyethyl methacrylate [16].

In our work, we use a separate crosslinking mechanism to inter-connect hydrogel-based building blocks on a macroscopic as well as microscopic scale. The here presented mechanism is based on the well-known dimerization of maleimides undergoing a photochemical [2+2] cycloaddition [17,18,19]. The benefit of this method is the facile UV-induced radical copolymerization of DMMI-functionalized acrylamide (DMMIAAm) with acrylic monomers and the reliable and robust inter-connection of maleimide units, such that the two processes can be temporally separated from each other utilizing different UV sensitive initiators. Here, we copolymerize acrylamide (AAm, formulation G1a and G1b) and *N*-isopropylacrylamide (NIPAAm, formulation G2) with *N*,*N*-methylenebisacrylamide (BIS-AAm) and DMMIAAm upon UV exposure to form stable hydrogels initiated via lithium phenyl-2,4,6-trimethylbenzoylphosphinate (LAP). Then, illuminating in the same wavelength region—now by immersion in a solution containing the triplet sensitizer sodium thioxanthone-2,7-disulfonate (TXS)—the gels are inter-connected by triggering the dimerization of the maleimides [19,20]. We illustrate the utility of this mechanism by irreversibly inter-connecting millimeter-sized cubic as well as micrometer-sized spherical hydrogel building blocks yielding free-standing hydrogel assemblies.

## 2. Results and Discussion

**Figure 1:**
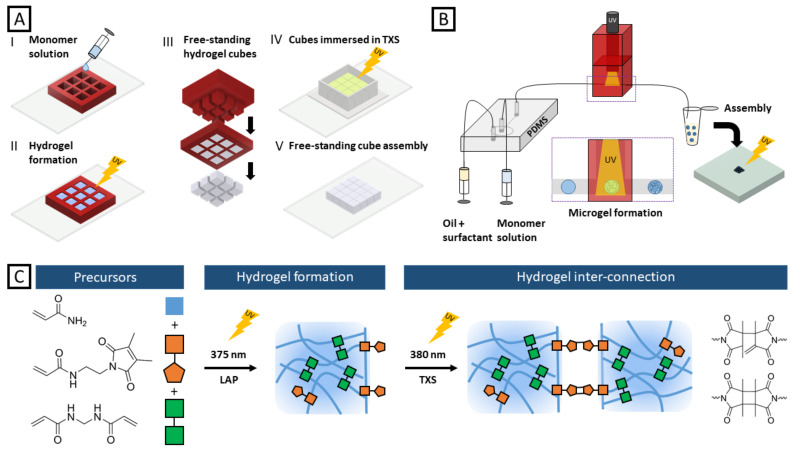
(**A**) Scheme of the experimental procedure to fabricate millimeter-sized hydrogel cubes. (I) A 3D-printed grid was fixated on a glass slide, and the monomer solution was poured into its voids. (II) The filled grid was illuminated by UV light to initiate polymerization. (III) The cured cubes were removed from the grid by a stamp. (IV) The cubes were immersed in a TXS solution, placed in a transparent 3D-printed chamber attached to a glass slide, and exposed to UV light again, inducing the inter-connection of building blocks via DMMI dimerization. (V) The resulting cube assembly was freely movable and could be transferred to water from the chamber. (**B**) For microfluidic microgel fabrication, a PDMS-based flow-focusing device was used. The produced emulsions were directly polymerized in the tubing connected to the outflow port when they passed the UV-illuminated area in the lamp fixation device. After collection and purification, the microgels were assembled in chambers and inter-connected. (**C**) Exemplary overview of the hydrogel formation utilizing AAm (blue), DMMIAAm (orange), and BIS-AAm (green). After UV-induced hydrogel formation initiated by LAP with an reported absorption maximum of 375 nm [21], DMMI groups remained available for a second crosslinking step. DMMI dimerization occurred after addition of TXS with an absorption maximum of 380 nm and a second UV exposure, yielding two dimerization products [22] and, thus, chemical microgel and building block inter-connection, respectively.

The concept of hydrogel preparation as well as their subsequent crosslinking is depicted in Figure 1. First, the aqueous monomer solutions consisting of either formulation G1 (AAm, DMMIAAm, BIS-AAm, and LAP) or formulation G2 (NIPAAm, DMMIAAm, BIS-AAm, and LAP) were poured into a 3D-printed grid with cubic void dimensions of 3 mm × 3 mm × 3 mm that is attached to a glass slide (Figure 1A). After UV irradiation at 365 nm for 10 min in the case of AAm and for 20 min in the case of NIPAAm as a monomer, respectively, the hydrogel structures were removed from the grid utilizing a stamp with similar dimensions. Thus, to inter-connect the cubic building blocks, they were immersed in an aqueous solution containing 0.1 M TXS confined by a chamber hosting a maximum of 16 cubes. The inter-crosslinked hydrogel arrays were then transferred to a petri dish or onto a glass slide for further experiments. In Figure 1B, the microfluidic fabrication of spherical hydrogel building blocks made from formulation G1a (Table 1, cf. 4.5) is illustrated. Here, we used a PDMS-based flow-focusing device with the outflow tubing being connected to a home-made, 3D-printed “lamp fixator”, ensuring reproducible, constant UV energy exposure among different microfluidic experiments.

After purification, the resulting hydrogel microparticles were poured into a confining chamber and subsequently inter-crosslinked in a 0.2 M TXS solution. Direct DMMI dimerization occurs upon UV irradiation at a wavelength around 235 nm, being highly energetic and potentially harming the polymer backbone of the hydrogel building blocks [23]. As TXS is activated at similar wavelengths as where LAP is decomposing with an absorption maximum of 375 nm [21], the same UV light source can be used to realize both consecutive steps: hydrogel formation and inter-crosslinking of the respective hydrogels (Figure 1C). When TXS absorbs UV light around 380 nm [20] the molecule is excited to its singlet state. It subsequently undergoes inter-system crossing to its triplet state and excites the DMMI molecule by energy transfer, initiating the dimerization reaction of the latter [22]. Seiffert and coauthors previously utilized TXS and DMMI-functionalized acrylamide to fabricate hydrogel networks and provided an in-depth study of the dimerization mechanism. In that study, it was discovered that in aqueous solution the dimerization reaction rather yields asymmetric products than the cyclobutane ring products, and crosslinking efficiency ranging from 16% to 62% dependent on the concentration of DMMI-bearing polymer [22]. Later, Seiffert et al. also exploited the TXS-initiated dimerization of DMMI to form thermo-responsive core–shell particles for controlled release via droplet microfluidics [24].

### 2.1. Inter-Connected PAAm Arrays

**Figure 2:**
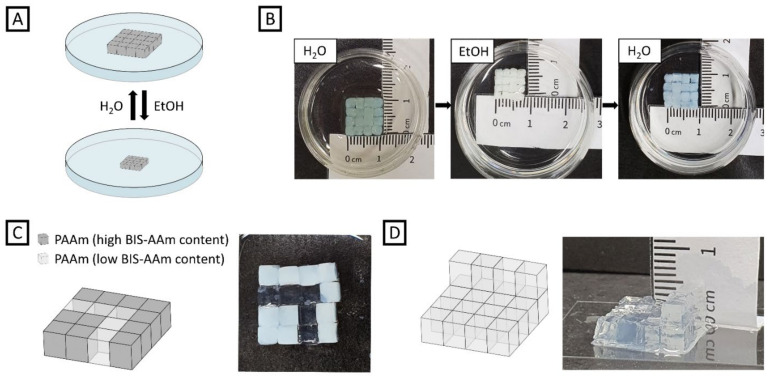
(**A**) Experimental setup for investigating inter-crosslinking stability of a PAAm-based cube array during swelling in different solvents. (**B**) Photographs of the swelling experiment of an opaque PAAm-based cube array. For deswelling, the as-fabricated hydrogel array was immersed in ethanol for 24 h, and, subsequently, immersed in DI water for another 24 h for swelling. (**C**) Illustration (left) and photograph (right) of a preliminary diffusion cell composed of opaque PAAm-based cubes with high BIS-AAm content and transparent PAAm-based cubes with low BIS-AAm content. (**D**) Graphic illustration (left) and photograph (right) of a multilayered, 3D PAAm-based cube array.

As introduced above, we make use of DMMI upon free-radical polymerization to form hydrogel networks. We first determined the solubility limit of DMMIAAm in DI water to be at approx. 34 mg mL^−1^, which equals a fraction of DMMI in the solid content of about 9.0 mol% in the hydrogel formulation G1a. Thus, the variability in inter-connection strength is limited for aqueous systems, although a higher DMMI content should lead to a higher dimerization rate, and therefore a stronger interaction between the hydrogel-based building blocks. Still, we chose the DMMI content to be 7.5 mol% for the three formulations, G1a, G1b, and G2 (Table 1). Figure 2B shows inter-crosslinked hydrogel building blocks made of G1a yielding mechanically stable arrays that resist shaking in a water-filled petri dish, for instance. To quantify the array’s stability upon motion, we mounted the petri dish onto a thermo shaker (MKR 13, Hettich Benelux, Geldermalsen, The Netherlands). The hydrogel array withstood applied rotation speeds up to 700 rpm for more than 5 min. For higher rotation speeds we observed increasing disintegration of the particle assembly. Further, the array was transferred onto a microscopy glass slide, keeping its structure. The transfer is conducted carefully, as the inter-connection of the cubes is sensitive to bending of the array. In addition to agitation tests, the mechanical stability was examined by swelling experiments. For that, the inter-connected array was placed in an ethanol bath for 24 h and thereafter placed in a water bath for another 24 h (Figure 2A, B). As illustrated in Figure 2B, the hydrogel array collapsed in ethanol. Despite the large deformation with a volume shrinkage of 60%, the array remained inter-crosslinked and could be moved in the petri dish. Subsequent placement in water led to the array’s swelling with a volume increase of 116% back to its initial state. During swelling, the array still remained undamaged and was stable against agitation. Working towards complex materials, we connected building blocks with varying mass content of BIS-AAm, yielding transparent (formulation G1b, Table 1) and opaque (formulation G1a, Table 1) hydrogel blocks (Figure 2C). Here, the cubes were assembled precisely to create a pathway that mimics a diffusion cell. In there, the transparent, less crosslinked cubes could transport larger molecules, while the surrounding, more crosslinked cubes would block larger molecules. To prove that the inter-connection solely relies on the DMMI dimerization instead of inter-block adhesion or crosslinking of unconsumed acrylamide groups, we first UV-irradiated an array of acrylamide-based hydrogel blocks without DMMI moieties under the same irradiation conditions as for the DMMI-functionalized, acrylamide-based hydrogel blocks. We observed an instant collapse of the array by only gently pushing the building blocks. Since there was no inter-connection withstanding these small forces, we subsequently immersed the same non-functionalized hydrogel building blocks in a solution of LAP and UV-irradiated the array again for 10 min to potentially trigger the reaction of unsaturated acrylamide moieties. Again, no inter-connection was observed, indicating that the interaction of building blocks rather relies on the dimerization of DMMI than on free-radical polymerization of excess acrylic moieties.

Apart from only forming two-dimensional array sheets consisting of one layer of building blocks, the stacking of building blocks in z-direction and subsequent crosslinking leads to three-dimensional hydrogel constructs, which is especially interesting for a potential application in additive manufacturing. In our experiments, the number of stackable layers is limited by the height of the confinement chamber and the hydrogel’s optical density in our experiments. Thus, we built a transparent 3D object with the cubes made of hydrogel formulation G1b being placed at two different levels to illustrate the applicability of our approach for future 3D printing relying on successive layer stacking of particle-based formulations (Figure 2D). As the success of DMMI dimerization depends on UV intensity, it was not possible to fabricate a multilayer 3D object composed of opaque, optically dense PAAm cubes due to light loss by absorption.

### 2.2. Inter-Connected Thermo-Responsive PNIPAAm Arrays

**Figure 3:**
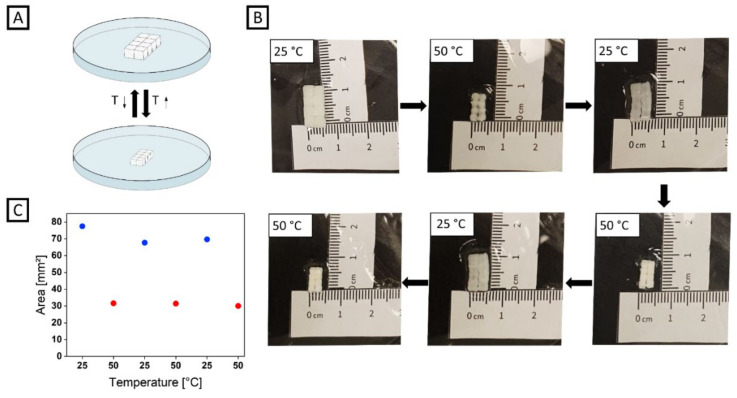
(**A**) Experimental setup for investigating inter-crosslinking stability of a PNIPAAm-based cube array towards temperature-dependent swelling. (**B**) Photographs of the swelling experiment of an opaque PNIPAAm-based cube array. For deswelling, the as-fabricated hydrogel array was immersed in a water bath and the temperature was raised to 50 °C. For subsequent swelling, the water bath was cooled to 25 °C. This cycle was repeated twice. (**C**) Change in area of a PNIPAAm-based cube array in water as a function of temperature. The blue data points indicate the area at 25 °C, the red data points indicate the area at 50 °C.

For fabricating thermo-responsive hydrogel arrays, NIPAAm was chosen as the precursor material. As PNIPAAm is thermo-responsive, undergoing a phase transition when reaching its lower critical solution temperature (LCST) at around 32 °C in aqueous media, we utilized exactly this behavior to determine the stability of DMMI dimerization against large deformations originating from triggered swelling and deswelling (Figure 3A). In Figure 3B, an inter-connected array of PNIPAAm-based building blocks prepared from formulation G2 (Table 1) is shown. The array exhibits similar stability as the afore-mentioned PAAm-based array when being gently agitated. The array was immersed in water, and the temperature was raised to 50 °C. Upon phase transition and, thus, deswelling of the PNIPAAm network, the array’s area significantly shrunk by 60%. Cooling down below the LCST to 25 °C led to the swelling of the hydrogel building blocks and their polymer network with an increase in area by 117% (Figure 3C). The heating and cooling cycle was repeated twice, with the array remaining robustly inter-connected after each step. After all cycles were completed, the array could still be moved without breaking apart.

### 2.3. Inter-Connected PAAm-Based Microgels

**Figure 4:**
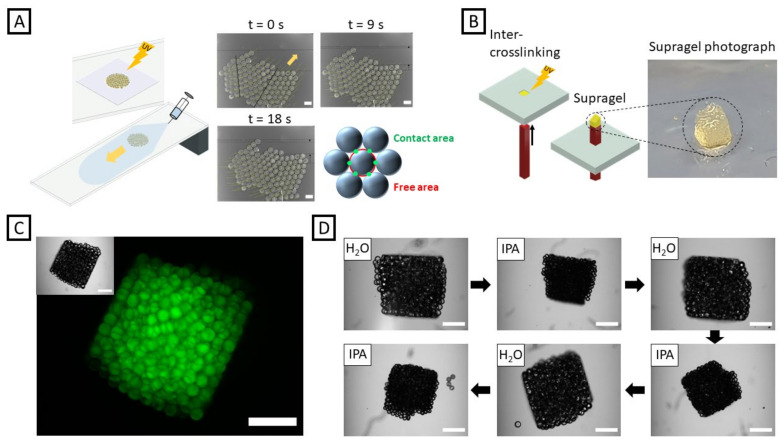
(**A**) Investigating the stability of inter-connected microgel sheets based on hydrogel formulation G1c by flow-induced motion. Motion images were recorded at t = 0 s, t = 9 s, and t = 18 s. The microgels coordinated in a hexagonal lattice, only exhibiting six contact points to neighbors (green) with the red lines indicating the surface regions that are not in contact with neighboring particles. The scale bars denote 200 µm. (**B**) Microgels stacked in a UV-transparent chamber to trigger DMMI dimerization upon UV irradiation and building block crosslinking before transfer to water. (**C**) Bright-field (inset) and fluorescence microscopy images of a supragel casted following the approach in (**B**). The scale bars denote 500 µm. (**D**) Supragel immersed in H_2_O and repeatedly de-swollen in isopropyl alcohol. The supragel retained its overall structure after three contraction and expansion cycles, with only single particles disconnecting. The scale bars denote 500 µm.

The functionality and potential system integration of a 4D material also relies on the local resolution where a respective property is localized. Micrometer-sized building blocks can be formed via droplet-based microfluidics with microgel diameters obtainable in the sub-100 µm range. Droplet-based microfluidics offers full control over the size and property of each single microgel with a variety of base materials applicable. Here, we microfluidically fabricated DMMI-functionalized acrylamide-based microgels to investigate the utilization of dimerization-based inter-crosslinking also on the microscale. As depicted in Figure 4A, monolayers of microgels made out of formulation G1c were fabricated in a thin reaction chamber. After removing the chamber, the glass slide below was tilted and carefully flushed with water. The microgel monolayer remaining on that glass slide was stable upon flow-induced motion, while unconnected microgels would spread over the glass slide. To produce a three-dimensional supragel, a transparent 3D-printed chamber (made from the commercial resin Clear Impact) with dimensions of 1.5 mm × 1.5 mm × 1.5 mm was filled with microgels exhibiting an average diameter of 142 ± 4 µm. The supragel was punched out by a small stick and transferred to a petri dish. As shown in Figure 4B, the supragel’s appearance is not perfectly cubic, with one side being cut off at a certain angle due to insufficient inter-crosslinking of microgels in this area. This may be due to the fact that the microgels, as indicated in Figure 4C,D, are opaque and, thus, the UV light might not reach all sections of the chamber uniformly. Further, the microgels exhibit adhesion towards surfaces like glass, leading to higher disruptive forces created upon removal of the supragel from the preparation chamber. Still, the supragel at hand exhibits rectangular shape at two sides (Figure 4C) as well as sharp edges, indicating that the approach is applicable towards a variety of geometries. To evaluate the stability of the supragel, the structure was repeatedly immersed for 2 min in DI water and deswollen in isopropyl alcohol thereafter (Figure 4D). Here, the area of the analyzed side decreased from A = 2.06 mm^2^ to A = 1.09 mm^2^ (−47%) in the first deswelling step. Upon re-immersion in DI water, the cube turned on a different side, exhibiting an area of A = 1.93 mm^2^. A second immersion in IPA resulted in an area decrease to A = 1.11 mm^2^ (−42%). The third swelling-deswelling cycle resulted in a change from A = 1.93 mm^2^ to A = 1.19 mm^2^ (−38%). Although some particles were disconnected during the experiment, the cubic construct is generally stable upon repeated expansion and contraction. The disconnection rather appeared when re-immersing the supragel in DI water, which caused the gel to flip over and being agitated in the solution due to the high surface tension of water on polystyrene.

## 3. Conclusions

In here, we investigated the UV-induced DMMI dimerization to inter-connect hydrogel building blocks towards polymer supragels. First, we studied the applicability of the approach for millimeter-sized, cubic structures with flat surfaces formed from acrylamide. Sixteen blocks were successfully inter-connected into hydrogel sheets, being mechanically stable against shear forces in solution upon agitation experiments as well as during contraction–expansion cycles switching between water and ethanol. Further, two acrylamide-based hydrogel species exhibiting different crosslinking degrees and, thus, different optical properties were inter-connected to create a hydrogel-based prototype of a guiding system with spatially tailored diffusivity. Second, we applied the same dimerization concept to form thermo-responsive PNIPAAm hydrogel assemblies, e.g., for future supragel-based sensor systems. Here, eight PNIPAAm-based building blocks were successfully connected, and the stability of the cube layer was investigated by three cycles of temperature-induced deswelling and swelling of the inter-connected array, with the array being still intact after repeated actuation. While the fabrication of a millimeter-sized cubic construct is rather straight forward due to their flat interfaces, we also investigated the feasibility of DMMI dimerization for spherical microgels fabricated via droplet-based microfluidics. Although the contact area is rather low due to the hexagonal arrangement of these spheres, we demonstrated the fabrication of stable microgel monolayers as well as three-dimensional cubic-like supragels that remained mostly stable after three cycles of swelling and deswelling switching between water and isopropyl alcohol. The future fabrication of micrometer-sized cubic structures should improve the overall stability of corresponding assemblies due to the increase in contact area between the microscopic building blocks. Further experiments need to be carried out to optimize process parameters, e.g., UV exposure energy, TXS, and DMMI concentration. Additionally, two different types of base materials, e.g., AAm and NIPAAm could be used to create more complex supragels with combined properties in one building block assembly. With the studied inter-connection mechanism and used building blocks being scalable from micro- to millimeter size, our approach may contribute to the development of novel additive manufacturing techniques based on hydrogel particle-based materials.

## 4. Materials and Methods

All chemicals were used without further purification unless stated otherwise. Anhydrous sodium carbonate, acrylamide (AAm, ≥99%), acryloyl chloride, magnesium sulfate, dimethylsulfoxide-d6, chloroform-d3, deuterium oxide, barium chloride dihydrate (≥99.999%), calcium carbonate (reagent plus), fluorescein isothiocyanate-dextran (FITC-dextran, M_W_ = 2,000,000), *N*,*N*-methylenebisacrylamide (BIS-AAm, ≥99.5%), phenolphthalein (ACS reagent), sulfuric acid fuming (puriss. p.a., 20% SO_3_), thioxanthen-9-one (97%) were purchased from Sigma-Aldrich (St. Louis, MO, USA. Lithium phenyl-2,4,6-trimethylbenzoylphosphinate (LAP, >98%), trifluoroacetic acid, triethylamine, and 2,3-dimethylmaleic anhydride were purchased from TCI chemicals (Tokyo, Japan). *N*-isopropylacrylamide (NIPAAm, 99%) and sulfuric acid (96%) were purchased from Acros Organics. 3M^TM^ Novec^TM^ 7500 (HFE 7500, >99%) was purchased from IoLiTec (Heilbronn, Germany). *N*-tert-butyloxycarbonyl-1,2-ethylenediamine was purchased from Carbolution Chemicals (St. Ingbert, Germany). Dichloromethane, chloroform, ethyl acetate, *n*-hexane, and acetone were purchased from Fisher Scientific (Hampton, VA, USA). Sodium chloride was purchased from Carl Roth (Karlsruhe, Germany). Clear Impact (Liqcreate, Utrecht, The Netherlands)was purchased from 3DJake (Paldau, Austria). Perfactory^®^ R11 was purchased from EnvisionTEC (Gladbeck, Germany).

Deionized (DI) water with a resistance of 18.2 MΩ cm was prepared in a Milli-Q Direct 8 water purification system (Merck Millipore, Burlington, MA, USA).

^1^H-NMR spectra were recorded on a Bruker Avance III 500 (Bruker, Billerica, MA, USA; equipment located in Dresden, Germany).

3D-printed parts were designed with Autodesk^®^ Inventor and fabricated with an ASIGA PICO 2 HD (Asiga, Alexandria, Australia). 3D printing of grid structures and corresponding stamps made of R11 for later building block fabrication was conducted at a set layer thickness of 25 µm and an exposure energy of 60 mJ cm^−2^ per layer. Open chambers made of Clear Impact for hydrogel cube assembly and microgel assembly were printed at a layer thickness of 25 µm and an exposure energy of 55 mJ cm^−2^ per layer. The 3D-printed parts were cleaned with IPA and post-cured for 200 s in a photopolymerization chamber (Otoflash G171, NK Optik, Baierbrunn, Germany).

**Figure 5:**
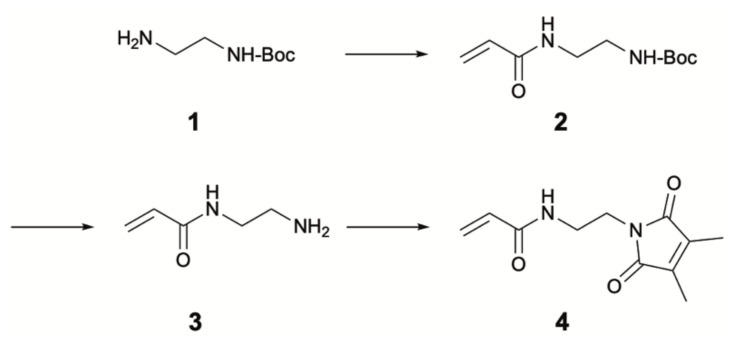
Synthesis of DMMI-functionalized acrylamide.

The synthesis of DMMIAAm was performed according to Ref. [25] in three steps as outlined in Figure 5.

### 4.1. Synthesis of N-Tert-butyloxycarbonyl-N’-acryl-1,2-diaminoethane (***2***)

Anhydrous sodium carbonate (4.9 g, 46 mmol) was added to a solution of *N*-tert-butyloxycarbonyl-1,2-ethylenediamine (7.4 g, 46 mmol) in dichloromethane (50 mL). While being continuously stirred and cooled in an ice/sodium chloride bath, a solution of acryloyl chloride (4.2 g, 46 mmol) in dichloromethane (20 mL) was dropped slowly into this solution over 1 h. Then, the reaction mixture was stirred at room temperature overnight. After filtration, the filtrate was dried with magnesium sulfate, and the solvent was removed in vacuo to give **2** as a white solid. The powder yield was 9.6 g (98%), and the structure was confirmed via ^1^H-NMR spectroscopy.

### 4.2. Synthesis of N-(2-Aminoethyl)acrylamide (***3***) as TFA Salt

Trifluoroacetic acid (TFA, 100 mL) was added to a solution of **2** (9.6 g, 45 mmol) in dichloromethane (30 mL), and the mixture was stirred for 1 h at room temperature. The solvent was removed in vacuo to give **3** as TFA salt in quantitative yield. The structure was confirmed via ^1^H-NMR spectroscopy.

### 4.3. Synthesis of N-[2-(3,4-Dimethyl-2,5-dioxo-2,5-dihydro-pyrrol-1-yl)ethyl]acrylamide (DMMIAAm) (***4***)

To obtain the free base **3**, **3**-TFA was dissolved in chloroform (60 mL), and the pH of the solution was adjusted to 8–9 by the addition of triethylamine. Subsequently, a solution of 2,3-dimethylmaleic anhydride (4.7 g, 37 mmol) in chloroform (120 mL) was added dropwise. The resulting reaction mixture was stirred at 50 °C under argon for 24 h. After that, the mixture was filtered, washed with water (4 × 200 mL), and dried with magnesium sulfate. The solvent was removed in vacuo, and the residue was purified by recrystallization from hexane/ethyl acetate (1:1) to obtain **4** as a white solid. The final powder yield was 3.3 g (33%, related to 2,3-dimethylmaleic anhydride), and the structure was confirmed via ^1^H-NMR spectroscopy.

### 4.4. Synthesis of Sodium Thioxanthone-2,7-disulfonate (TXS)

The synthesis of TXS was adopted from Ref. [20]. In short, 5 g thioxanthen-9-one was dissolved in 100 mL fuming sulfuric acid and refluxed for 3.5 h at 110 °C. The solution was cooled down, poured on 300 g ice, and neutralized by adding CaCO_3_, until CO_2_ production stopped. Precipitated CaSO_4_·2H_2_O was filtered off, and the filtrate was isolated. An aqueous solution (20 mL) containing 5 g of BaCl_2_·2H_2_O was slowly added to the yellow filtrate under stirring. The precipitated barium salt was recrystallized three times in H_2_O yielding yellow, needle-shaped crystals. The barium salt was then dissolved in hot H_2_O, and Na_2_CO_3_ mixed with phenolphthalein was added until a weak red coloring appeared. The precipitated BaCO_3_ was filtered off, and the filtrate was reduced under vacuum to approx. 15 mL. After adding 0.01 M H_2_SO_4_ dropwise, the solution was filtered again, and residual H_2_O was removed utilizing a rotary evaporator. The resulting crystals of TXS were washed multiple times with acetone and dried in an oven at 130 °C for three hours. The yield was 0.48 g. The aromatic structure was confirmed via ^1^H-NMR spectroscopy (500 MHz, DMSO-d6): δ = 8.72 ppm (d, 2H), δ = 7.97 ppm (dd, 2H), and δ = 7.83 ppm (d, 2H).

The TXS content of the salt was calculated from two samples via ^1^H-NMR spectroscopy to be approx. 90–97% (*w*/*w*) with acetonitrile as internal reference (δ = 2.08 ppm, s, 3H).

### 4.5. Fabrication of AAm-Based and NIPAAm-Based Hydrogel Cubes

For all prepared hydrogel cubes, the secondary crosslinker DMMIAAm (crosslinker 2) was dissolved in DI water at 50 °C under vigorous shaking. Afterwards, the monomer AAm and NIPAAm, respectively, the primary crosslinker BIS-AAm (crosslinker 1) and the photoinitiator LAP, were added to the solution and dissolved under further shaking. The respective formulations are summarized in Table 1. Until usage, the mixtures were stored in the dark to prevent polymerization.

To fabricate hydrogel cubes, the respective formulation was filled into a custom-designed grid that is fixed on a glass slide. The grid is designed with overall dimensions of 15 mm × 15 mm × 3 mm. The cubic voids are designed to be 3 mm × 3 mm × 3 mm. The stamp consists of a base plate with dimensions of 15 mm × 15 mm × 5 mm with the positive cuboid structures designed on top exhibiting dimensions of 2.7 mm × 2.7 mm × 3.5 mm.

For UV-initiated polymerization, the grid was positioned under a UV-source (Nu-8, 8 W, 365 nm, Herolab, Wiesloch, Germany) and irradiated for 10 min in the case of AAm as a monomer and for 20 min in the case of NIPAAm as a monomer, respectively, at a fixed distance of 0.5 cm. Afterwards, the hydrogel cubes were removed from the grid utilizing the matching stamp and immersed in DI water. Formulation G1a and G2 resulted in opaque hydrogel cubes, whereas formulation G1b resulted in transparent hydrogel cubes.

### 4.6. Microfluidic Device Fabrication and Setup Design

For the fabrication of water-in-oil (W/O) emulsions, microflow cells made of poly(dimethylsiloxane) (PDMS) were fabricated utilizing conventional photo- and soft lithography. The master structures with a target channel height of 150 µm were fabricated by spin-coating the negative photoresist SU-8 2050 (Micro Resist Technology, Berlin, Germany) onto a three-inch silicon wafer (Siegert Wafer, Aachen, Germany). A mask aligner (MJB3, Süss MicroTec, Garching, Germany) was used to define the desired microchannel structure by a printed photomask. The non-hardened photoresist was removed with a developer (mr-Dev600, Micro Resist Technology, Berlin, Germany). The microfluidic channel dimensions were designed to be 150 µm for the dispersed phase, 150 µm for the continuous phase, and 250 µm for the droplet-transporting channel. Subsequently, the microfluidic master device was replicated in PDMS by replica molding. The PDMS base and crosslinker mixture (Sylgard^®^ 184 silicone elastomer kit, Dow Corning, Midland, MI, USA) were mixed at a ratio of 10:1, degassed in a planetary centrifugal mixer (ARE-250, Thinky, Laguna Hills, CA, USA), poured onto the respective master device, and cured for 2 h at 65 °C. Afterwards, the PDMS replica was cut out from the mold and peeled off from the master. Inflow and outflow ports were punched with a biopsy punch (1.0 mm diameter, KAI Medical, Solingen, Germany). Finally, the microchannel-containing side of the PDMS replica was bonded to a microscopy glass slide (76 × 52 mm) via oxygen plasma treatment (80 W for 15 s, MiniFlecto 10, Plasma Technology, Herrenberg, Germany). Prior to usage of the microfluidic device, the microchannels were hydrophobized by injecting a 1% (*v*/*v*) solution of (tridecafluoro-1,1,2,2-tetrahydrooctyl)trichlorosilane (Gelest, Morrisville, PA, USA) in Novec 7500 (IoLiTec, Heilbronn, Germany). To perform the microfluidic experiment, the microfluidic devices were connected to high-precision syringe pumps (Harvard Apparatus Pump 11 Pico Plus Elite, Harvard Apparatus, Holliston, MA, USA) via PE tubing (inner diameter: 0.38 mm, outer diameter: 1.09 mm, Hartenstein, Würzburg, Germany). A 1000 µL gastight syringe (1750 TLL SYR, Hamilton, Reno, NV, USA) was used for the dispersed phase (DP), and a 3 mL disposable syringe (BD Luer lock tip, Becton Dickinson, Franklin Lakes, NJ, USA) was used for the continuous phase (CP). The experiment was followed on an inverted bright-field microscope (Axio Vert.A1, Carl Zeiss, Oberkochen, Germany) equipped with a high-speed digital camera (Miro C110, Vision Research Inc., Wayne, NJ, USA).

### 4.7. General Procedure of Microgel Fabrication

For the fabrication of microgels, the DP consisting of the monomer solution with 1449.8 mM AAm, 158.6 mM MBAAm, 130.0 mM DMMIAAm, and 3.4 mM LAP (formulation G1c), was injected at 800 µL hr^−1^ and emulsified into droplets by the CP consisting of a triblock copolymer surfactant (PFPE-PEG-PFPE, RAN Biotechnologies, Beverly, MA, USA, 2% *w*/*w*) in Novec 7500 at a flow rate of 85 µL hr^−1^. For UV polymerization, the outflow tubing was connected to a 3D-printed, custom-built lamp fixation device made of R11 and illuminated by a UV source (OmniCure^®^ S1500, 200 W, 250 to 450 nm, 44.6 mW cm^−2^, Asslar, Germany) at a fixed distance of 2 cm. The emulsion droplets were collected at intervals of 30 min in Eppendorf tubes. For purification, the microgels were transferred into water by washing with 20% (*v*/*v*) 1*H*,1*H*,2*H*,2*H*-perfluoro-1-octanol (PFO, Sigma-Aldrich, St. Louis, MO, USA) in Novec 7500 three times. Bright-field, wide-field fluorescence, and confocal microscopy imaging of the emulsion droplets and microgels was conducted on a TCS SP8 microscope (Leica, Wetzlar, Germany). To evaluate the resulting microgels size, 100 microgels were measured manually using the software ImageJ [26]. The resulting diameters were measured to be 210 ± 5 µm for the corresponding microgels, while, after dimerization, the particle size decreased to 147 ± 3 µm. These particles were used to create single microgel layers.

### 4.8. Inter-Crosslinking of DMMI-Functionalized Hydrogel Cubes and Microgels

The fabricated hydrogel cubes from Chapter 4.5 were placed in an open chamber with dimensions of approx. 12 mm × 12 mm × 3.3 mm to obtain a 4 × 4 array with the cubes being in direct contact with each other. For inter-connection, an aqueous solution supplemented with 0.1 M TXS was added on top of the arranged hydrogel cubes. After a short incubation for 5 min, the hydrogel array was exposed to the UV source (OmniCure^®^ S1500) for 5 min at a fixed distance of 2.5 cm. Finally, the inter-connected array was carefully transferred into a DI water-filled petri dish.

The fabricated microgels from Chapter 4.7 were pipetted into an imaging spacer chamber (SecureSeal^TM^, Grace Bio-Labs, Bend, OR, USA) with a diameter of 9 mm and a height of 0.12 mm. The solvent was carefully removed and replaced by an aqueous solution supplemented with 0.05 M TXS. After a short incubation for 5 min, the hydrogel array was exposed to a UV source (Nu-8, 8 W, 365 nm, Herolab, Wiesloch, Germany) for 5 min at a fixed distance of 1.5 cm. Subsequently, the inter-connected arrays were carefully transferred into a DI water-filled petri dish.

For fabricating a three-dimensional microgel cube, microgels with 13.8% (*w*/*w*) AAm, 3.0% (*w*/*w*) BIS-AAm, 2.2% (*w*/*w*) DMMIAAm, and 0.2% (*w*/*w*) LAP were used. The hydrogel microparticles with a mean diameter of 142 ± 4 (n = 100) µm were repeatedly immersed and stacked in a 3D-printed chamber made of Clear Impact with designed chamber dimensions of 1.5 mm × 1.5 mm × 1.5 mm attached to a glass slide. After the chamber was filled with particles, 2 µL of a 0.2 M TXS solution was added on top, and the filled particle chamber was placed in an UV chamber (Type DR-301C, 36 W) for 5 min. Afterwards, the 3D-printed chamber was removed from the glass slide and the supragel was pushed out of the chamber by a small, 3D-printed stick fitting into the void. The supragel was then immersed in DI water for imaging and swelling experiments.

## Figures and Tables

**Table 1:** Summary of formulations used for hydrogel cube fabrication.

Sample	Monomer	Crosslinker 1	Crosslinker 2	Photoinitiator	Ʃ [% (*w*/*w*)]
G1a	AAm, 1449.8 mM	127.3 mM	128.0 mM	3.4 mM	15.2
G1b	AAm, 1449.8 mM	58.1 mM	123.0 mM	3.4 mM	14.0
G2	NIPAAm, 1450.0 mM	44.3 mM	122.0 mM	3.4 mM	19.9

## Data Availability

The data presented in this work are available on request from the corresponding author.
